# Bioresponsive Materials for Drug Delivery Based on Carboxymethyl Chitosan/Poly(γ-Glutamic Acid) Composite Microparticles

**DOI:** 10.3390/md15050127

**Published:** 2017-04-28

**Authors:** Xiaoting Yan, Zongrui Tong, Yu Chen, Yanghe Mo, Huaiyu Feng, Peng Li, Xiaosai Qu, Shaohua Jin

**Affiliations:** School of Material Science and Engineering, Beijing Institute of Technology, Beijing 100081, China; yanxt911218@163.com (X.Y.); zrtong1994@163.com (Z.T.); bitmoyanghe@163.com (Y.M.); 18640109501@163.com (H.F.); lpfightforever@163.com (P.L.); shawnqu@foxmail.com (X.Q.); jinshaohua@bit.edu.cn (S.J.)

**Keywords:** carboxymethyl chitosan, poly(γ-glutamic acid), microparticle, drug release

## Abstract

Carboxymethyl chitosan (CMCS) microparticles are a potential candidate for hemostatic wound dressing. However, its low swelling property limits its hemostatic performance. Poly(γ-glutamic acid) (PGA) is a natural polymer with excellent hydrophilicity. In the current study, a novel CMCS/PGA composite microparticles with a dual-network structure was prepared by the emulsification/internal gelation method. The structure and thermal stability of the composite were determined by Fourier transform infrared spectroscopy (FTIR), X-ray powder diffraction (XRD), scanning electron microscope (SEM), X-ray photoelectron spectroscopy (XPS), and thermogravimetric analysis (TGA). The effects of preparation conditions on the swelling behavior of the composite were investigated. The results indicate that the swelling property of CMCS/PGA composite microparticles is pH sensitive. Levofloxacin (LFX) was immobilized in the composite microparticles as a model drug to evaluate the drug delivery performance of the composite. The release kinetics of LFX from the composite microparticles with different structures was determined. The results suggest that the CMCS/PGA composite microparticles are an excellent candidate carrier for drug delivery.

## 1. Introduction

In the past decades, various drug delivery systems have been developed to improve the releasing behaviors and effectiveness of drugs, and lower their side-effects. An ideal drug delivery system should be able to increase drug solubility, provide a sustained release system to avoid rapid breakdown and excessive use, and improve biodistribution [1]. Mild reaction conditions and absence of toxic or/and active organic substances are required to maintain the activity of active drugs, such as proteins [2,3]. Controlled-release systems based on smart bioresponsive materials have been extensively studied to delivery drugs [4]. The intraluminal pH of the gastrointestinal tract should be taken into consideration for the design of drug delivery system. The oral drug undergoes exposure to stomach (pH 1.0–2.5), duodenum (pH 6), and terminal ileum (pH 7.4) environments. Drugs encapsulated in pH-sensitive carriers should survive the harsh microenvironment in the stomach prior to entering the small intestine [5,6]. In addition, an ideal drug carrier should be biocompatible and biodegradable [7,8].

Chitosan is a biocompatible, biodegradable, and antibacterial polysaccharide. Chitosan and its derivatives are promising candidates of drug carrier and have been applied in many biomedical fields [9]. However, the chemical modification of chitosan requires toxic organic substances and chitosan is poorly soluble in water, which limits its encapsulation ability of hydrophilic drugs [10,11]. Therefore, various chitosan derivatives were reported as drug carriers with improved hydrophilicity and swelling behavior [12,13,14,15]. The compositions of chitosan with hydrophilic polymers, such as alginate and poly (acrylic acid), is an ideal strategy to develop drug carriers with desired swelling behaviors [16,17].

Poly(γ-glutamic acid) (PGA) is a hydrophilic polypeptide that can be used as a carrier of various drugs and biomolecules, such as proteins, growth factors, and genes, and its degradation product is nutritional to cellular proliferation [16,17,18,19,20,21]. Many chitosan/PGA composites, such as nanoparticles [22,23,24,25,26], hydrogels [27], and microcapsules [28], have been reported as drug carriers. Yu et al. [29] prepared carboxymethyl chitosan/poly(γ-glutamic acid) nanoparticles and used it as the carrier of bovine serum albumin. The introduction of PGA enhanced the incorporation ability of chitosan with biological molecules and intracellular trafficking. The composite nanoparticles exhibited a long-acting release of protein drugs in hyper-gluconic acid. However, extensive studies indicate that the covalent crosslink of chitosan has at least two drawbacks: (1) potential irritation to mucosal membranes due to the toxic crosslinker residues, such as glutaric dialdehyde; and (2) possible reactions between drugs and the reactive crosslinkers, such as genipin [30,31]. To overcome these limits, tripolyphosphate, citrates, and metal ions (e.g., Ca^2+^) have been used for the ionic crosslinking of chitosan-based drug carriers in recent studies [32,33].

In the present work, PGA was composited with carboxymethyl chitosan (CMCS) to improve the hydrophilicity of CMCS. The CMCS/PGA composite microparticles were gelled using Al^3+^ as the crosslinker under mild conditions. Such ionic crosslinking with the metal ion as the crosslinker is favorable to the scaled-up industrial production, and highly sensitive to external stimulation. Hydrophilic drug levofloxacin (LFX) was encapsulated as a model drug to evaluate the encapsulation effectiveness of the CMCS/PGA composite microparticles. The controlled and sustained drug release behaviors of the composite microparticles were determined by its swelling behaviors and in vitro drug release experiments.

## 2. Results and Discussion

### 2.1. Characterization of CMCS/PGA Composite Microparticles

**Figure 1:**
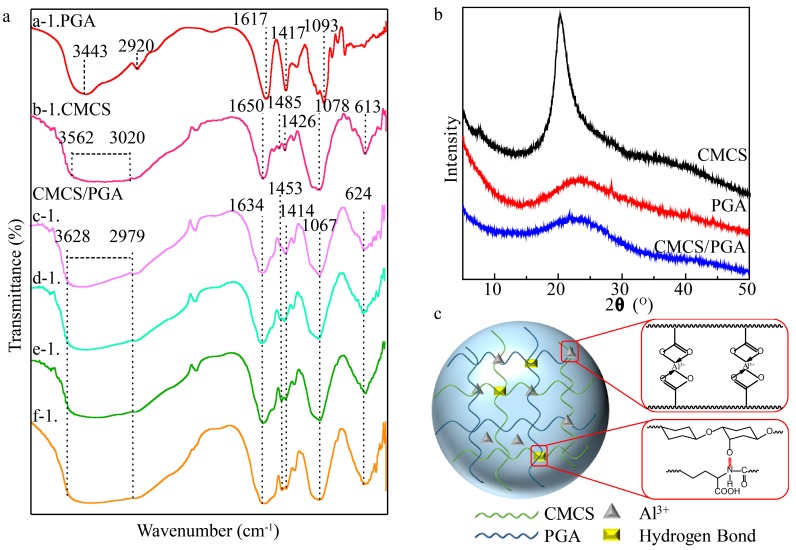
Structural characterization of CMCS/PGA composite microparticles. (**a**) FT-IR spectra of PGA (a-1), CMCS (b-1) and CMCS/PGA composite microparticles prepared at the feeding ratio m_CMCS_:m_PGA_= 9:1 (c-1), 8:2 (d-1), 7:3 (e-1), 6:4 (f-1); (**b**) XRD patterns of CMCS, PGA and CMCS/PGAcomposite microparticles; and (**c**) the scheme of the composite microparticles.

The Fourier transform infrared spectroscopy (FTIR) spectra of CMCS, PGA, and CMCS/PGA composite microparticles prepared at different feeding ratios are shown in Figure 1a. The characteristic absorption peaks of PGA at 3443 cm^−1^ and 2920 cm^−1^ were ascribed to the O-H and N-H stretching vibrations, respectively, and the absorption peak at 1617 cm^−1^ was attributed to the asymmetric stretching vibration of C=O. The absorption peak at 1417 cm^−1^ was assigned to the N-H stretching vibration of amide [34]. CMCS exhibited an absorption band at 3020–3562 cm^−1^ that was attributed to the O-H and N-H stretching vibrations. Its absorption peaks at 1650 cm^−1^, 1485 cm^−1^, 1426 cm^−1^, and 1078 cm^−1^ were ascribed to the C=O asymmetric stretching vibration, N-H (amide II band) bending vibration, N-H stretching vibration, and C-O-C bonds stretching vibration [35], respectively.

Compared with CMCS, CMCS/PGA composite microparticles exhibited a broader absorption bond in the range of 2972–3628 cm^−1^ and the absorption peak of the N-H stretching vibration shifted from 1426 cm^−1^ to 1414 cm^−1^, indicating that the hydrogen bond between the polymer chains was enhanced [36]. The COO- peaks blueshifted from 1650 cm^−1^ to 1634 cm^−1^ as CMCS composited with PGA and Al^3+^. Both C-O-C peaks of CMCS at 1078 cm^−1^ and PGA at 1126 cm^−1^ shifted to 1067 cm^−1^ as the composite formed. These results indicated that the carboxyl group was successfully coordinated with Al^3+^ [37], which was further confirmed by the characteristic absorption peak of Al-O at 624 cm^−1^ in the IR spectrum of CMCS/PGA composite microparticles [38].

Figure 1b shows the X-ray diffraction (XRD) patterns of CMCS, PGA, and CMCS/PGA microparticles. The intensity and position of a diffraction peak can reflect the crystalline degree of a the structure. CMCS exhibited a crystal structure with a crystalline diffraction peak at 20°. No diffraction peak was found in PGA, indicating that it was amorphous. The weak diffraction peak of CMCS/PGA microparticles indicates that the crystalline degree of the CMCS in CMCS/PGA composite microparticles was significantly destructed due to the amorphous PGA interpenetrating into CMCS polymer chains, the coordination between the carboxyl group and Al^3+^, and the hydrogen bond formed between the carboxyl group and amide or amino group.

**Figure 2:**
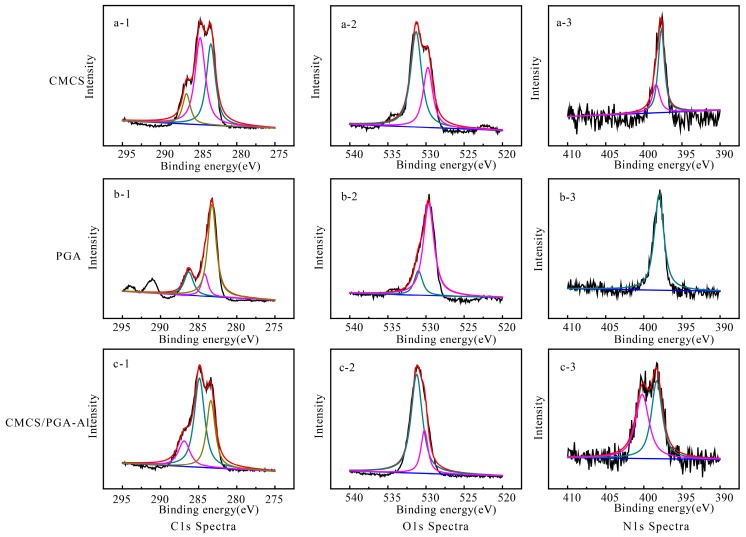
XPS spectra of CMCS, PGA, and CMCS/PGA composite microparticles. The spectra include C1s (a-1, b-1, c-1), O1s (a-2, b-2, c-2), and N1s (c-1, c-2, c-3); a-1,2,3 belong to CMCS, b-1,2,3 belong to PGA, c-1,2,3 belong to CMCS/PGA composite microparticles.

The composite microparticles were further characterized with XPS. Figure 2 shows the C1s, O1s and N1s XPS spectra of CMCS, PGA, and CMCS/PGA. The corresponding binding energies and the relative contents of C, N, and O elements can be found in Supplementary Table S1. The binding energy of O1s revealed that the hydroxyl groups on the surface of CMCS/PGA composite microparticles (531.2 eV) shared similar binding energy with those of CMCS (531.3 eV) and PGA (531.0 eV). The binding energy of C=O on CMCS/PGA composite microparticles (530.2 eV) is higher than those of CMCS (529.8 eV) and PGA (529.7 eV), indicating the carboxyl group was successfully coordinated with Al^3+^, is consistent with the FT-IR results.

The binding energy of the N-H in CMCS/PGA composite microparticles (400.2 eV) is higher than that in CMCS (398.4 eV), and the binding energy of C-N in CMCS/PGA composite microparticles (398.3 eV) is higher than those in CMCS (397.8 eV) and PGA (398.0 eV), indicating that hydrogen bonds were formed between the carboxyl group and amide or amino group. These results suggest that a double network structure of CMCS/PGA composite microparticles was formed by the coordination between carboxyl group and Al^3+^, and the hydrogen bond between carboxyl group and amide or amino group (Figure 1c).

### 2.2. Morphology of CMCS/PGA Composite Microparticles

**Figure 3:**
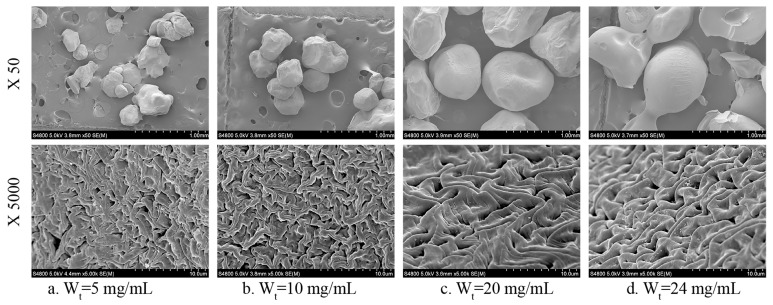
SEM images (50×) and (5000×) of CMCS/PGA microparticles prepared at different polymer concentrations (W_t_).

The morphology of CMCS/PGA composite microparticles prepared with different concentrations of CMCS and PGA were imaged by scanning electron microscope (SEM) at the magnifications of 50× and 5000×. As shown in the low magnification SEM images in Figure 3, the composite was comprised of spherical microparticles with a narrow size distribution, and the particle size increased with the increase of polymer concentration. The high magnification SEM images revealed a porous surface morphology on the microparticles. Such porous structure can promote a swelling behavior, drug release and response to external stimulation, and provide the ion channels to maintain the balance between the osmotic pressures inside and outside of the microparticles.

### 2.3. Swelling Behaviors of CMCS/PGA Composite Microparticles

**Figure 4:**
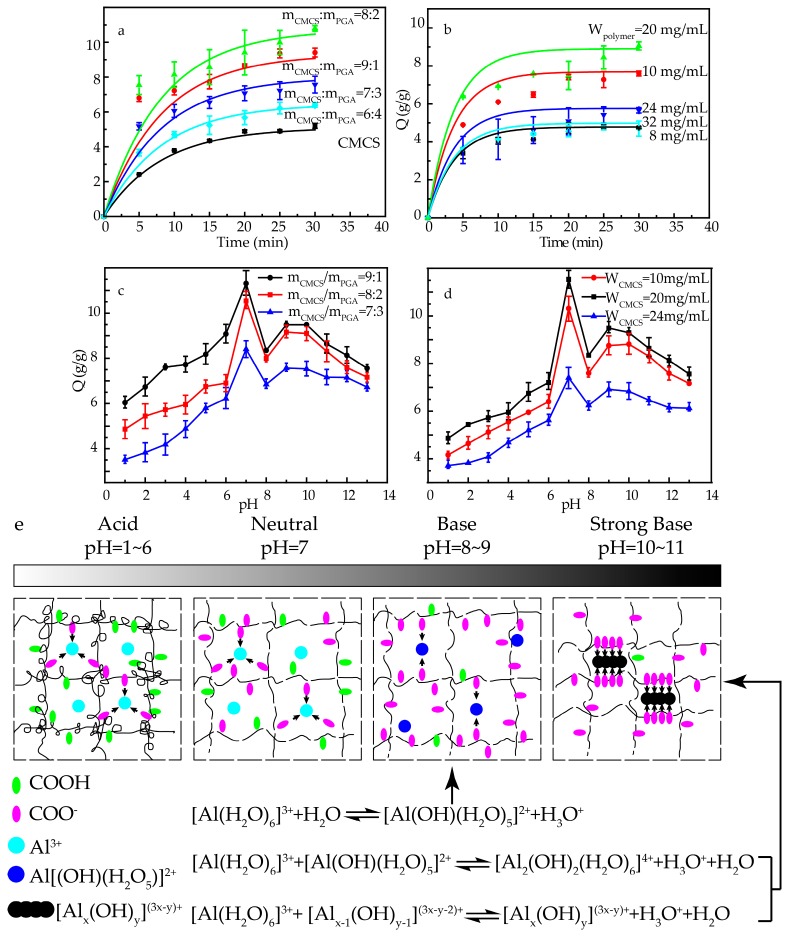
The swelling behaviors and pH responsive behaviors of CMCS/PGA composite microparticles and the proposed mechanism of the pH responsive behavior. (1) Effects of feeding ratio (**a**) and CMCS weight ratio (**b**) on the swelling behavior of CMCS/PGA composite microparticles; (2) Effects of feeding ratio (**c**) and CMCS weight ratio (**d**) on the pH responsive behavior of CMCS/PGA composite microparticles; and (3) the proposed mechanism of pH responsive behavior (**e**).

The CMCS/PGA composite microparticles prepared under different conditions exhibited different swelling properties due to their different hydrophilicity, osmotic pressures between inside and outside of the microparticles, and structures. Therefore, the feeding ratio and total polymer concentration of CMCS and PGA were optimized to obtain desired swelling behavior of CMCS/PGA composite microparticles. Figure 4a,b shows the swelling kinetic curves of CMCS/PGA composite microparticles prepared under different conditions. The Voigt model fitted the experimental data well, indicating the swelling behavior of CMCS/PGA composite microparticles could be simulated as polymer creep motion.

As shown in Figure 4a and Table 1, all CMCS/PGA composite microparticles prepared under different conditions exhibited higher swelling rates and ratios than CMCS microparticles, indicating that the introduction of PGA improved the hydrophilicity of CMCS. The swelling ratio of CMCS/PGA composite microparticles increased with PGA content, peaked at m_CMCS_:m_PGA_ = 8:2, and decreased as PGA content increased further. The highest swelling rate was also obtained with the composite prepared at m_CMCS_:m_PGA_ = 8:2. It can be explained that the hydrophilicity of CMCS/PGA composite microparticles increases with PGA content due to its rapid and high water absorption. Water is introduced into the microparticles rapidly, resulting in excellent swelling behaviors. However, extremely high PGA content can strengthen the intermolecular interaction and enhanced the physical tangle of the double network, which inhibits water permeation, and thus reduce the swelling ratio and rate.

The total polymer concentration of CMCS and PGA was then varied to determine its effects on the swelling behavior of the CMCS/PGA composite. As shown in Figure 4b, both the swelling ratio and rate increased first, peaked, and decreased with the increase in total polymer concentration. SEM imaging indicated that the composite particles prepared with low polymer concentrations were relatively small. The intermolecular entanglement between CMCS and PGA at low concentrations mainly results from hydrogen bonding and, thus, is weak, leading to low crosslink density and incomplete double network. Increasing the polymer concentration can improve the swelling behavior of CMCS/PGA composite microparticles. However, higher polymer concentration results in larger particles, which decreases the specific area for water contact and increases the intermolecular entanglement between CMCS and PGA. Water is then inhibited to permeate into CMCS/PGA composite microparticles, which decreases the swelling ratio and rate.

The swelling behaviors of CMCS/PGA composite microparticles at different pHs were investigated and the results were shown in Figure 4c,d. All tested CMCS/PGA composite microparticles showed similar pH responses. The swelling ratio increased with the increase of pH, peaked at pH = 7 and significantly declined as the pH increased to 8. The swelling ratio slightly increased as pH varied from 9 to 10. Further increasing pH led to lower swelling ratios.

The responsive swelling mechanism of CMCS/PGA composite microparticles to pH is summarized in Figure 4e. In acid condition, the significant amount of H^+^ in a strong acid solution can decrease the dissociation degree of –COOH, which weakens the repulsion between –COO^−^ groups. The polymer chains were rolled and the network of CMCS/PGA composite microparticles shrank, which inhibited the swelling of the composite and resulted in low swelling ratios. Increasing pH can increase the dissociation degree of –COOH, leading to stronger repulsion between –COO^−^ groups. The polymer network swelled up due to the high repulsion and hydrophilicity of –COO^−^ and amide bonds, which improved the swelling behavior of the composite. The swelling ratio reached the maximum at pH = 7. In basic condition, [Al(OH)(H_2_O)_5_]^2+^ was formed as pH further increased, which decreased the amount of coordination sites with COO^−^ and COOH. The decreased crosslink density led to a significantly low swelling ratio at pH = 8. In strong base (the pH increased to 9–10), the dissociation degree of –COOH reached the maximum and the repulsion between –COO^−^ groups was maximized, which dominated the swelling behavior again. The swelling ratio was slightly increased. At pH = 11, the massive Na^+^ shielded COO^−^ groups, which weakened the repulsion force. Meanwhile, aluminum was hydrolyzed to polyaluminium [Al_x_(OH)_y_]^(3x−y)+^ forming a highly-crosslinked network with the polymers [39], which further decreased the swelling ratio.

### 2.4. Hydrophilic Properties of CMCS/PGA Composite Microparticles

**Figure 5:**
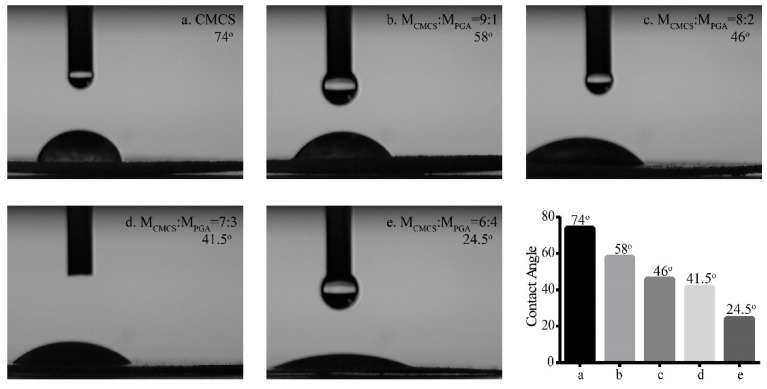
Water contact angles of CMCS microparticles (**a**) and CMCS/PGA composite microparticles prepared at m_CMCS_:m_PGA_ = 9:1 (**b**), 8:2 (**c**), 7:3 (**d**), and 6:4 (**e**).

The hydrophilicity of CMCS/PGA composite microparticles prepared at different feeding ratios was determined by their water contact angles. All CMCS/PGA composite microparticles exhibited water contact angles less than 90°, smaller than that of CMCS microparticles (Figure 5), indicating that the introduction of PGA improved the hydrophilicity of CMCS. In addition, the water contact angle decreased with the increase of PGA content, further demonstrating that PGA enhanced the hydrophilicity of CMCS.

### 2.5. Drug Release Behaviors of CMCS/PGA Composite Microparticles

**Figure 6:**
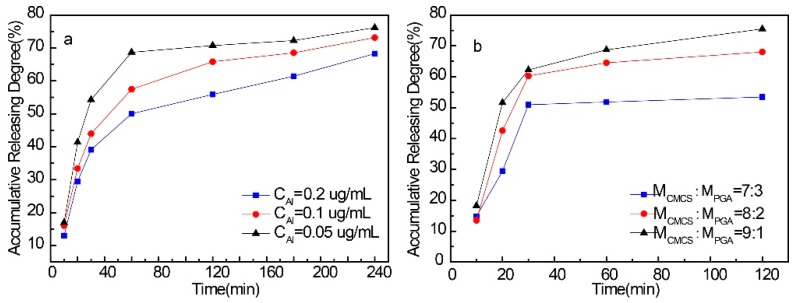
The drug release behaviors of LFX loaded CMCS/PGA composite microparticles prepared at different [Al^3+^] levels (**a**) and different feeding ratio (**b**).

The drug encapsulation and release behaviors of CMCS/PGA composite microparticles were investigated using LFX as a model drug. The highest LFX encapsulation efficiency of the composite was obtained as 28.6% (*w*/*w*). The Al^3+^ concentration and feeding ratio for the composite preparation exhibited significant effects on its drug release behavior (Figure 6). More than 70% LFX was released in one hour from the loaded composite microparticles prepared at low [Al^3+^] concentrations, which was not ideal for a drug carrier (Figure 6a). Increasing [Al^3+^] for the composite preparation significantly improved the sustained release behavior and extended the maximum drug release time to 4 h. It can be explained that a higher [Al^3+^] results in a denser inner network, which encapsulates LFX more tightly. The release kinetics were simulated with a zero-order kinetic model, one-order dynamic model, and a Higuchi model, respectively. The results are shown in Table 2. When [Al^3+^] was less, the release of LFX was better fitted with the Higuchi model. At this time, because the crosslinking density in the microparticles was lower, the mass transfer resistance of the drug in the network was weaker and easy to spread out from the network. Then the drug on the surface of the microparticles was dissolved in the liquid and drugs within the microparticles were gradually diffused to the surface of microparticles and eventually spread into the liquid due to the concentration gradient. With the increase of the [Al^3+^], the release model was gradually deviated from the Higuchi model and turned to one-order kinetics. Then, with increased [Al^3+^], the network density was increased. As a result, the resistance of the drug from the microparticles was increased and its release was controlled by surface diffusion and dissolution mechanisms. In addition, Table 2 indicates that using the same equation to perform the simulation, the slope of the curve obtained was decreased with the increase of [Al^3+^]. This also demonstrated that the increase of [Al^3+^] may result in a decreased release rate of LFX from the microparticles.

In addition, the sustained release time increased with the increase of PGA content in the CMCS/PGA composite (Figure 6b) due to the enhanced hydrogen bond and intermolecular entanglement.

## 3. Materials and Methods

### 3.1. Materials

Carboxymethyl chitosan (CMCS, 1000 kDa) was synthesized according to a method reported in the literature [40]. The substitution degree of the carboxymethyl was determined as 0.75 by elemental analysis. Poly(γ-glutamic acid) (PGA, >92.0%, 1000 kDa) was purchased from Nanjing Saitaisi Biotechnology Co., Ltd. (Nanjing, China); Aluminum sulfate was purchased from Tianjin Fu Chen Chemical Reagent Factory (Tianjing, China); Levofloxacin (LFX, 98%) was purchased from Shanghai Macklin Biochemical Co., Ltd. (Shanghai, China).

### 3.2. Preparetion of CMCS/PGA Composite Microparticles

A polymer solution of CMCS and PGA at a certain concentration was mechanically stirred, and added to an aluminum sulfate solution at a tested concentration dropwise under stirring. The suspension was stirred for 1 h, suction filtrated, and dried to afford CMCS/PGA composite microparticles. The feeding ratio, total polymer concentration of CMCS and PGA, and [Al^3+^] were varied to optimize the preparation condition of CMCS/PGA composite microparticles. The detailed preparation conditions are shown in Table 3.

LFX was loaded in CMCS/PGA composite microparticles as follows: 0.8 g LFX was dissolved in a polymer solution of CMCS and PGA. The mixture was added to a 0.1 g/mL aluminum sulfate solution dropwise. The suspension was stirred for 1 h, suction filtrated, and dried to form LFX-loaded CMCS/PGA composite microparticles.

### 3.3. Characterization of CMCS/PGA Composite Microparticles

#### 3.3.1. Fourier Transform Infrared Spectroscopy (FTIR) Analyses

Fourier Transform Infrared Spectroscopy (FTIR) analyses were performed on a Thermo-Nicolet NEXUS 470 Spectroscopy (ThermoFish, Waltham, MA, USA) equipped with a KBr beam splitter. The test samples were prepared with KBr pellets.

#### 3.3.2. XRD Diffractograms Analyses

XRD diffractograms were recorded in the 2θ range of 5.0°–80.0° on a Rigaku D/Max-1200 instrument (Rigaku, Tokyo, Japan) equipped with Ni-filtered Cu Kα radiation (40 kV, 40 mA) to determine the crystallinity of Alg/PGA composite microparticles.

#### 3.3.3. X-ray Photoelectron Spectroscopy (XPS) Analyses

X-ray Photoelectron Spectroscopy (XPS) analyses were conducted on a PHI QUANTERA-II instrument (Ulvac-PHI Inc., Chigasaki, Kanagawa, Japan) equipped with a monochromatized Al KRX-ray source operated at 25 W and 15 kV. For wide-scan spectra, an energy range of 0–1100 eV was used with a pass energy of 280.00 eV and a step size of 1.00 eV. High-resolution spectra were collected at a 26.00 eV pass energy using a step size of 0.025 eV. The XPS results were further fitted in a nonlinear least squares curve fitting program (XPS-peak-41 software, Version 4.0).

#### 3.3.4. Surface Analysis via Scanning Electron Microscopy

SEM was used to examine the structure and surface morphology of the produced microparticles. Microparticles were dusted onto a double-sided tape on an aluminum stub, coated with a gold layer using a gold sputter coater and imaged on an S-4800 SEM instrument (Hitachi, Tokyo, Japan) with a 5 kV electron beam.

### 3.4. Determination of the Swelling Behavior of Composite Microparticles

The swelling behavior of the composite microparticles was investigated by the filtering bag test method. A sample (0.2 g) was put in a nylon bag and immersed into the liquid to be absorbed at room temperature. The mass of the swollen sample was weighed every 5 min after the excess water was removed. The water uptake ratio (*Q*) of the microparticles were calculated as the following equation:

(1) $$Q=\left(\left(W_{1}-W_{0}-W_{2}\right)/W_{0}\right)\times 100\%$$

where, *W*_1_ is the weight of the test sample and bag at a given swelling time, *W*_2_ is the weight of the nylon bag and *W*_0_ is the initial weight of the sample. Every group of samples was tested three times, and the mean and SD of swelling ratio was calculated.

The swelling process of the composite microparticles is similar to the creeping response of the polymer. It is caused by the combination of hydrophilic interactions, the repulsion force between the anions, and the osmotic pressures between the inside and outside of the networks. Therefore, the swelling kinetic parameters of the composite microparticles can be fitted by the Voigt model [40]. Assuming that the force *σ*_0_ is applied to the model at time *t*_0_ and the corresponding response *ε* was produced at time *t*, the model can be expressed as the following equation:

(2) $$\varepsilon \left(t\right)=\sigma_{0}/E\left\{1-\exp\left[\left(t_{0}-t\right)/\tau_{0}\right]\right\}=\varepsilon (\infty )\left\{1-\exp\left[\left(t_{0}-t\right)/\tau_{0}\right]\right\}$$

where, *τ*_0_ is the relaxation time that is theoretically inversely proportional to the swelling ratio of the microparticles and *E* is the Young’s modulus that represents the resistance to deformation. σ_0_/*E* equals to *ε*(∞), representing the maximum swelling ratio. The slope of forward straight part of the curve (*k_i_*) with *Q* = 0.7*ε*(∞) and the time (*t_c_*) when the swelling become slow can be calculated accordingly. The swelling rate is directly proportional to *k_i_* and inversely proportional to *t_c_*.

### 3.5. Water Contact Angle Measurement

The water contact angle (WCA) was measured with a drop-shape analysis system (KRUSS DSA100, Hamburg, Germany) at three different points for each sample. Three replicate specimens were tested for each sample and the relative standard deviation was within ±3%.

### 3.6. Pharmacokinetic Drug Release Analysis

The LFX stock solution (0.1 g/L) was diluted with 0.9% NaCl solution to 2, 4, 8, 16, 32, and 48 μg/mL. The absorbance of each standard at λ = 328 nm was measured using a TU-1810 UV-VIS system (Persee, Beijing, China) with 0.9% NaCl solution as the blank group to construct a standard curve of the LFX. The LFX concentration in the dialysate of loaded CMCS/PGA composite microparticles was determined by fitting its absorbance at λ = 328 nm to Equation (3).

To determine the drug release, 0.2 g LFX loaded CMCS/PGA composite microparticles were immersed in 100 mL 0.9% NaCl solution. Three milliliters of solution was taken at the predesigned time, measured for its absorbance at λ = 328 nm, and put back into the sample solution. The concentration of released LFX in the NaCl solution was calculated with Equation (3) to profile the pharmacokinetic drug release of the CMCS/PGA composite microparticles. The encapsulation efficiency and cumulative drug release was calculated with Equations (4) and (5) respectively.

(3) Absorbance=0.0288•[LFX]−0.0005(R=0.9994)

(4) $$\mathrm{Encapsulation}\;\mathrm{efficiency}\;(\%)=\left(\frac{\mathrm{Total}\;\mathrm{drug}\;\mathrm{used}-\mathrm{unencapsulated}\;\mathrm{drug}}{\mathrm{Total}\;\mathrm{drug}\;\mathrm{used}}\right)\;\times 100\%$$

(5) $$\mathrm{Cumulative}\;\mathrm{drug}\;\mathrm{release}\;(\%)=\left(\frac{\mathrm{Amount}\;\mathrm{of}\;\mathrm{drug}\;\mathrm{at}\;\mathrm{times}}{\mathrm{Total}\;\mathrm{drug}\;\mathrm{present}\;\mathrm{in}\;\mathrm{composite}\;\mathrm{microparticles}}\right)\;\times 100\%$$

The kinetics of LFX released from CMCS/PGA composite microparticles was determined by finding the best fit of the dissolution data (LFX-released fraction versus time) to distinct models: zero-order, first-order, and Higuchi [41,42].

Zero-order kinetics:

*y* = k_0*t*_ + *b* (6)

where *y* is the amount of LFX released at time *t*, and *k*_0_ is the zero-order release constant.

First-order kinetics:

*ln*(1 − *y*) = −*k*_1*t*_ + *b* (7)

where *y* is the amount of LFX released at time *t*, and *k*_1_ is the first-order release constant.

Higuchi’s model:

*y* = *k_H_t*^1/2^ + *b* (8)

where *y* is the amount of LFX released at time *t*, and *k_H_* is the Higuchi’s release rate constant.

## 4. Conclusions

CMCS/PGA composite microparticles were prepared by ionic crosslinking using Al^3+^ as the crosslinker. The microparticles exhibited the excellent properties of CMCS and high hydrophilicity of PGA. The FT-IR, XRD, and XPS analyses indicate that a double network structure was formed by the hydrogen bond between PGA and CMCS and the coordination between Al^3+^ and carboxyl groups. A systematic investigation was conducted to determine the effects of the PGA content and total polymer concentration of CMCS and PGA on the swelling behavior of the microparticles. The results revealed that the introduction of PGA significantly improved the hydrophilicity of CMCS microparticles. The composite preparation conditions for the highest swelling ratio and rate were optimized as: w_CMCS_ = 20 mg/mL and m_CMCS_:m_PGA_ = 8:2. In addition, the swelling behavior of the CMCS/PGA composite microparticles is pH sensitive, indicating that it is an ideal carrier for sustained drug delivery. The drug encapsulation and release behaviors were then investigated using hydrophilic LFX as a model drug. The LFX encapsulation efficiency of the microparticles reached as high as 28.6% (*w*/*w*), and an ideal sustained drug release was obtained. These results suggest that CMCS/PGA composite microparticles are a promising candidate carrier for drug delivery system.

## Figures and Tables

**Table 1:** Swelling kinetic parameter of various CMCS/PGA composite microparticles.

m_CMCS_:m_PGA_	σ_0_/E (g·g^−1^)		τ_0_ (min)	k_i_ (g·min^−1^)	t_c_ (min)
CMCS	5.105		7.712	0.383	9.394
9:1	9.316		7.333	0.740	8.788
8:2	10.738		6.355	0.987	7.576
7:3	8.006		7.308	0.637	8.788
6:4	6.462		7.897	0.478	9.394
C_CMCS+PGA_ (g/mL)		σ_0_/E (g·g^−1^)	τ_0_ (min)	k_i_ (g·min^−1^)	t_c_ (min)
0.008		4.786	6.513	0.426	7.879
0.010		7.690	6.338	0.708	7.576
0.020		8.902	5.355	0.974	6.354
0.024		5.763	5.589	0.602	6.667
0.032		4.985	6.239	0.463	7.576

**Table 2:** Simulation of the releasing kinetics of LFX loaded CMCS/PGA composite microparticles.

Conditions	Model	Fitting Equation	*R*
**C_Al_ = 0.05 μg/mL**	Zero-order equation	*y* = 0.0019*t* + 0.3971	0.7092
	One-order equation	ln(1 − *y*) = −0.0043*t* − 0.5346	0.8089
	Higuchi equation	*y* = 0.0389*t*^1/2^ + 0.2349	0.8192
**C_Al_ = 0.10 μg/mL**	Zero-order equation	*y* = 0.0020*t* + 0.3203	0.8292
	One-order equation	ln(1 − *y*) = −0.0044*t* − 0.3810	0.9072
	Higuchi equation	*y* = 0.0413*t*^1/2^ + 0.1535	0.9142
**C_Al_ = 0.20 μg/mL**	Zero-order equation	*y* = 0.0019*t* + 0.2706	0.8613
	One-order equation	ln(1 − *y*)= −0.0037*t* − 0.3038	0.9304
	Higuchi equation	*y* = 0.0388*t*^1/2^ + 0.1167	0.9317

**Table 3:** Conditions for preparation of various CMCS/PGA composite microparticles.

Samples	m_CMCS_:m_PGA_	Polymer Concentration (mg/mL)	[Al^3+^] (g/mL)
1	10:0	20	0.1
2	9:1	22	
3	8:2	25	
4	7:3	29	
5	6:4	33	
6	8:2	10	0.1
7		12.5	
8		25	
9		30	
10		40	
11	8:2	25	0.05
12			0.1
13			0.2

## Supplementary Materials

The following are available online at www.mdpi.com/1660-3397/15/5/127/s1, Table S1: The corresponding binding energies and the relative contents of C, N, and O elements in CMCS, PGA, and CMCS/PGA composite microparticles.
