# Supplementary material for: Bioresponsive Materials for Drug Delivery Based on Carboxymethyl Chitosan/Poly(γ-Glutamic Acid) Composite Microparticles
Source: Mar Drugs. 2017 Apr 28;15(5):127. doi: 10.3390/md15050127 (PMC5450533; doi:10.3390/md15050127)
Supplement: Supplementary file 1 [file marinedrugs-15-00127-s001.pdf]

Table S1. The corresponding binding energies and the relative contents of C, N, and O elements in CMCS, PGA, and CMCS/PGA composite microparticles.

	C1s		O1s		N1s	
**Samples**	**B.E.(eV)**	**Relative content**	**B.E. (eV)**	**Relative content**	**B.E. (eV)**	**Relative content**
CMCS	286.6 C=O	15.1%	531.3 C=O	64.0%	398.4 N-H	29.4%
	284.8 C-O	46.5%	529.8 C-O	36.0%	397.8 C-N	70.6%
	283.4 C-C,C-H	38.4%				
PGA	286.3 C=O	21.3%	531.0 C=O	19.2%		
	284.2 C-O	12.5%	529.7 C-O	80.8%	398.0 C-N	
	283.3 C-C,C-H	63.5%				
CMCS/PGA	286.9 C=O	17.5%	531.2 C=O	75.3%	400.2 N-H	50.5%
	284.9 C-O,	46.1%	530.2 C-O	24.7%	398.3 C-N	49.5%
	283.4 C-C,C-H	36.4%				
